# Construction of an Infectious DNA Clone of Grapevine Geminivirus A Isolate GN and Its Biological Activity in Plants Analyzed Using an Efficient and Simple Inoculation Method

**DOI:** 10.3390/plants13121601

**Published:** 2024-06-08

**Authors:** Can Liu, Shangzhen Yu, Jinying Wang, Yinshuai Xie, Hanwei Li, Xueqing Zhang, Chenlu Feng, Wenhao Zhang, Yuqin Cheng

**Affiliations:** Department of Pomology, College of Horticulture, China Agricultural University, Beijing 100193, China15754368031@163.com (J.W.); lhw_woaiguoshu@163.com (H.L.);

**Keywords:** grapevine geminivirus A, infectious viral clone, pathogenicity, inoculation method

## Abstract

The pathogenicity of grapevine geminivirus A (GGVA), a recently identified DNA virus, to grapevine plants remains largely unclear. Here, we report a new GGVA isolate (named GGVA^QN^) obtained from grapevine ‘Queen Nina’ plants with severe disease symptoms. The infectious clone of GGVA^QN^ (pXT-GGVA^QN^) was constructed to investigate its pathogenicity. *Nicotiana benthamiana* plants inoculated with GGVA^QN^ by agroinfiltration displayed upward leaf curling and chlorotic mottling symptoms. A simple, quick, and efficient method for delivering DNA clones of GGVA^QN^ into grapevine plants was developed, by which *Agrobacterium tumefaciens* cells carrying pXT-GGVA^QN^ were introduced into the roots of in vitro-grown ‘Red Globe’ grape plantlets with a syringe. By this method, all ‘Red Globe’ grape plants were systemically infected with GGVA^QN^, and the plants exhibited chlorotic mottling symptoms on their upper leaves and downward curling, interveinal yellowing, and leaf-margin necrosis symptoms on their lower leaves. Our results provide insights into the pathogenicity of GGVA and a simple and efficient inoculation method to deliver infectious viral clones to woody perennial plants.

## 1. Introduction

Viral diseases in viticulture lead to serious reductions in yield quantity and quality. With the development of high-throughput sequencing technology, new viruses infecting grapevines have been identified. To date, more than 80 different viruses from 17 families and 34 genera have been identified in grapevines [1,2]. Grapevine geminivirus A (GGVA) was first reported in 2017 using high-throughput sequencing [3]. Since its initial characterization, GGVA has also been reported in China [4], South Korea [5], New Zealand [6], and India [7]. GGVA is a monoparticle, single-stranded circular DNA virus belonging to the genus *Maldovirus* in the family *Geminiviridae*. The complete genome of GGVA ranges from 2903 to 2907 nucleotides (nt) in length [8], including two open reading frames (ORFs) on the viral sense strand encoding V1 (coat protein) and V2 (putative movement protein), and four ORFs on the complementary strand encoding C1 (replication-associated protein), C2 (transcriptional activator protein), C3 (replication enhancer), and C4 (host-activated protein) [3].

Infectious clones of viruses are important research tools for the analysis of viral biology and pathogenic mechanisms. Infectious clones of some grapevine viruses [8,9,10,11,12,13,14,15,16,17,18] have already been constructed. By using GGVA infectious clones, the pathogenicity of different GGVA isolates has been analyzed in *Nicotiana benthamiana* [8,16] and grapevine [16] plants. Those authors found that GGVA infection induces dwarfing and upward leaf curling symptoms in *N. benthamiana* plants, whereas grapevine plants agroinfiltrated with GGVA were asymptomatic [16].

Grapevine and other woody perennials are recalcitrant to mechanical inoculation. The main approaches for delivering infectious clones into grapevine plants include vacuum-assisted agroinfiltration [2,9,16] and agro-drenching [10,15,18]. However, these methods are complex and time-consuming, especially for the latter method, which requires more than 20 days to establish a viral infection. In addition, the two methods result in relatively low survival and infection rates for the treated plants [2]. Therefore, the development of an efficient and simple experimental system to launch grapevine infections is needed.

In the present study, we constructed an infectious DNA clone of GGVA isolate QN (pXT-GGVA^QN^), which was isolated from a grapevine ‘Queen Nina’ plant with severe disease symptoms, including malformation, upward curling of the leaf margins, vein clearing, and reduced leaf size. The biological activity of an infectious clone of GGVA^QN^ was analyzed in *N. benthamiana* and grapevine plants. In addition, we provide a simple, time-saving and highly efficient inoculation method to deliver an infectious clone of a virus into woody perennial plants, including grapevine.

## 2. Results

### 2.1. Construction of an Infectious Clone of GGVA Isolate QN

The recombinant plasmid pCE3-GGVA^QN^, containing the full genome of GGVA^QN^, was used as a template. The full genome of GGVA^QN^ (2905 nt) was 100% identical to the isolate we obtained from the grapevine ‘Marselan’ (GGVA^ML^, GenBank accession no. PP397181) (Supplementary Figure S1a), and shared 96.72% to 99.70% sequence identity with previous reported isolates. A phylogenetic tree was constructed based on the whole genome sequences of GGVA isolates from GenBank using the neighbor-joining method. We found that GGVA^QN^ is very close to the isolate reported by Sun et al. [8] (Supplementary Figure S1b).

**Figure 1:**
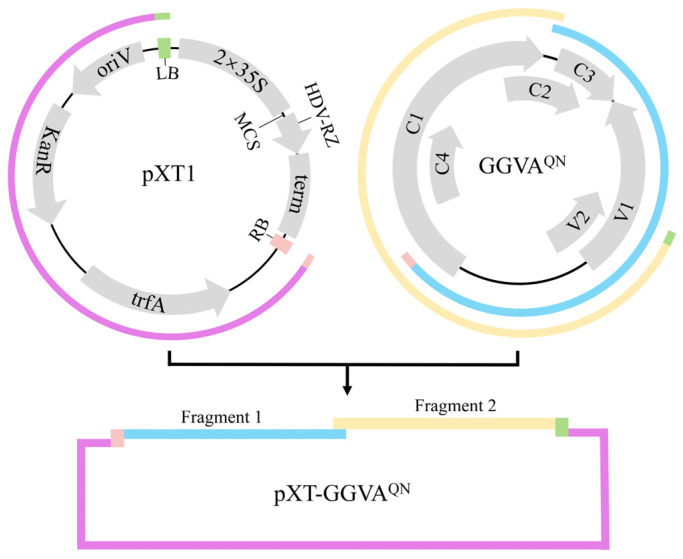
Diagram of the strategy used to build the infectious clone of grapevine geminivirus A isolate QN (pXT-GGVA^QN^). Fragment 1, fragment 2, and the linearized pXT1 are marked in blue, yellow, and purple, respectively. The 5′ terminus of fragment 1 and the 3′ terminus of fragment 2 separately contain the T-DNA right border (RB) and left border (LB), marked in pink and green, respectively. MCS, multiple cloning site region; oriV, origin of vegetative replication; KanR, kanamycin resistance gene; trfA, trans-acting replication protein that binds to and activates oriV; term: NOS terminator; HDV-RZ, hepatitis delta virus ribozyme; 2 × 35S, two copies of the 35S promoter region.

To construct an infectious clone of GGVA^QN^, two fragments covering 1.2× viral genome sequence amplified from pCE3-GGVA^QN^ and the linearized vector pXT1 (JN029690) [19] were assembled into circular plasmids (Figure 1), which were then transformed into competent *Escherichia coli* DH5α cells. Positive clones were identified by polymerase chain reaction (PCR), enzyme digestion, and sequencing. The recombinant plasmid (pXT-GGVA^QN^) was transformed into *Agrobacterium tumefaciens* GV3101.

### 2.2. Infectivity of GGVA^QN^ in N. benthamiana Plants

**Figure 2:**
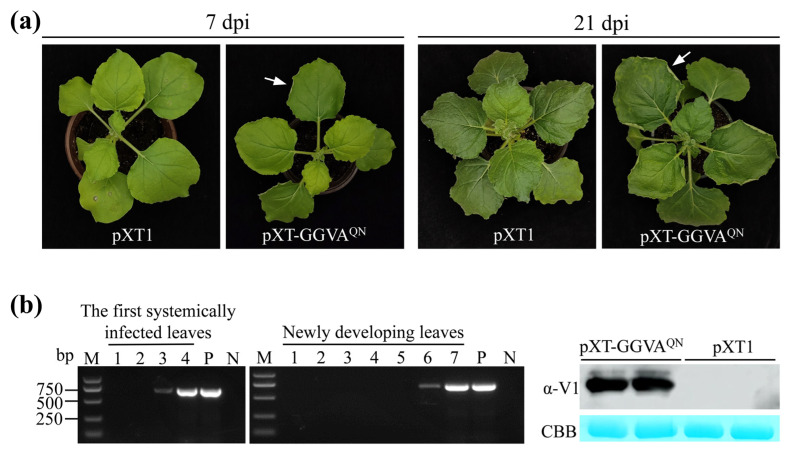
Systemic infection of GGVA^QN^ in *Nicotiana benthamiana* plants. (**a**) Symptoms induced by GGVA^QN^. An arrow in the left panel indicates upward curling of the leaf edge. (**b**) PCR (left and middle panels) and Western blot (right panel) assays confirm the presence of GGVA^QN^ in *N. benthamiana* plants. Lanes 1–7: samples collected 1 to 7 days postinfiltration (dpi), respectively. Upper newly developing leaves collected 6 dpi were used for detection of GGVA V1 protein by Western blotting with anti (α)-V1 antiserum. P and N: positive and negative controls with pXT-GGVA^QN^ and distillation-distillation H_2_O (ddH_2_O) instead of DNA, respectively. CBB, Coomassie brilliant blue staining confirmed equal loading.

Six-week-old *N. benthamiana* plants were agroinfiltrated with the infectious clone of pXT-GGVA^QN^, and agroinfiltration of the empty vector pXT1 served as a control. At 7 days postinfiltration (dpi), the pXT-GGVA^QN^-infiltrated leaves exhibited upward curling of the leaf edge, and symptoms of upward leaf curling together with chlorotic spots were observed on the upper systemically infected leaves at 21 dpi, whereas the control plants were asymptomatic (Figure 2a). All 37 *N. benthamiana* plants (10–13 plants, three repeats) were systemically infected with pXT-GGVA^QN^, as evidenced by PCR and Western blot assays showing the presence of GGVA^QN^ in the first systemically infected and upper new developing leaves of all GGVA^QN^-inoculated plants at 3 and 6 dpi, respectively (Figure 2b). These results indicated that pXT-GGVA^QN^ can successfully infect *N. benthamiana* plants and elicit upward leaf curling and chlorotic spot symptoms.

### 2.3. Infectivity of GGVA^QN^ in Grapevine Plants

Before inoculation assays, the virological condition of the in vitro-grown ‘Red Globe’ grapevine plantlets, which had tested negative for 19 grapevine viruses, as well as grapevine leafroll-associated virus 4 (GLRaV-4) strains GLRaV-5, GLRaV-6, GLRaV-9, GLRaV-De, and GLRaV-Pr, were resubjected to reverse transcription (RT)-PCR and PCR assays. They were all free of the targeted viruses (Supplementary Figure S2).

**Figure 3:**
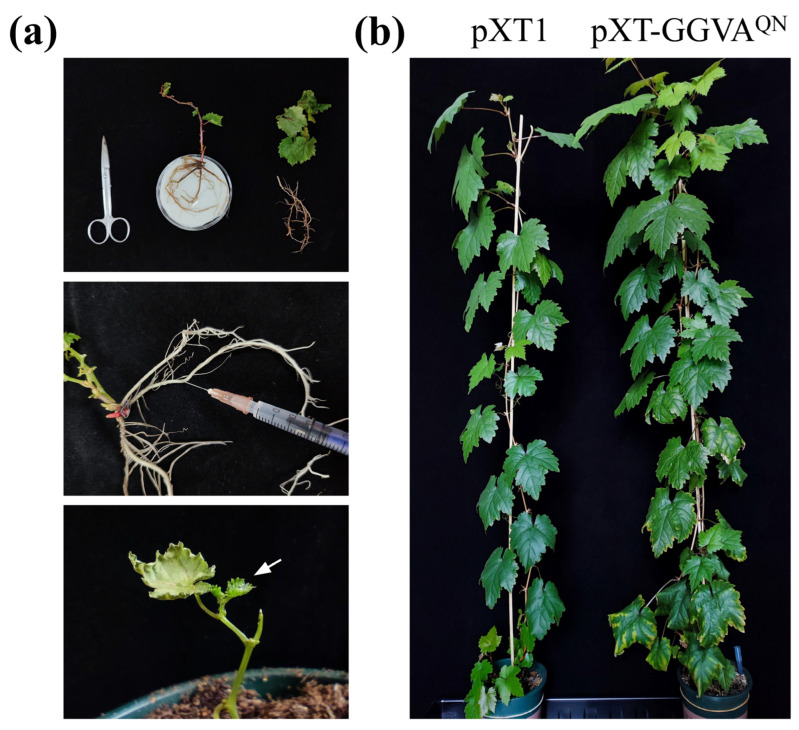
A simple, quick, and efficient method for delivering the infectious clone pXT-GGVA^QN^ into grapevine plants. (**a**) Diagram of the procedure for agroinfiltration. Arrow indicates the sprouting buds after inoculation and transplanting. (**b**) Symptoms induced by GGVA^QN^ in grapevine ‘Red Globe’ plants 4 months after inoculation and transplanting.

**Figure 4:**
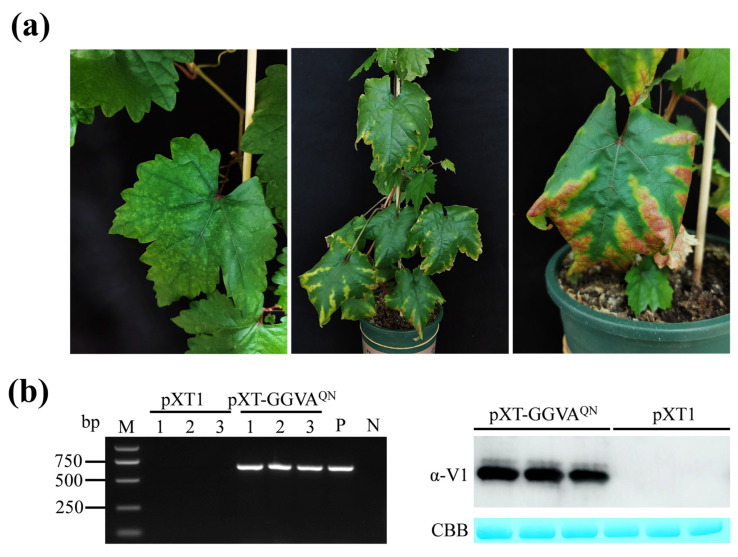
Systemic infection of GGVA^QN^ in grapevine ‘Red Globe’ plants. (**a**) Symptoms in upper (left panel) and lower (middle and right panels) leaves of grapevine plants inoculated with pXT-GGVA^QN^. Symptoms were recorded 2 (left and middle panels) and 5 (right panel) months after inoculation, respectively. (**b**) Systemic infection by GGVA^QN^ in grapevine ‘Red Globe’ plants was confirmed by PCR (left panel) and Western blot (right panel) assays. Samples were collected from three pXT-GGVA^QN^-inoculated and control plants 1 month after inoculation and transplanting, respectively. P and N: positive and negative controls with pXT-GGVA^QN^ and ddH_2_O instead of DNA, respectively. GGVA accumulation was detected with α-V1 antiserum. CBB, Coomassie brilliant blue staining confirmed equal loading.

We then used a very simple and efficient method to deliver pXT-GGVA^QN^ to grapevine plants. The roots of each in vitro-grown grapevine plantlet were directly inoculated with 0.5 mL of *Agrobacterium tumefaciens* cells carrying pXT-GGVA^QN^ by syringe and then transplanted into a pot (Figure 3a). The empty vector pXT1 served as a control. A total of 18 plants were inoculated with GGVA^QN^ in two repeat experiments (9 plants each), and 15 plants survived after transplanting. As shown in Figure 3b and Figure 4a, about 2 months later, ‘Red Globe’ plants inoculated with GGVA^QN^ showed chlorotic mottling on the upper leaves; symptoms of downward curling and necrosis of the leaf margins, and interveinal yellowing were also observed on the lower leaves. Five months later, the color of the interveinal tissues of the lower leaves of a few plants changed from yellow to red. All of these symptoms were absent in the corresponding control ‘Red Globe’ plants. All 15 surviving plants were systemically infected with GGVA^QN^, as confirmed by the presence of GGVA^QN^ in the upper leaves detected by PCR and Western blot assays 1 month after inoculation (Figure 4b). These results demonstrated that ‘Red Globe’ plants systemically infected with GGVA^QN^ display symptoms including chlorotic mottling, downward curling and necrosis of the leaf margins, and interveinal yellowing. Our results also indicated that the simple inoculation method developed in this study is efficient for launching the infection of grapevine.

## 3. Discussion

GGVA was found in samples from both grapevine ‘Nagano purple’ plants with leaf chlorotic ringspot symptoms and asymptomatic ‘Black Beet’ plants [3]. Fan et al. [4] reported that most grapevine samples that were positive for GGVA were asymptomatic, except for a few samples with foliar ringspot symptoms. Similarly, Jo et al. [5] reported the detection of GGVA in all 16 individual grapevine plants representing 12 different cultivars, but only the cultivar Shine Muscat displayed obvious symptoms (leaf malformation, vein clearing, and yellowing). Infectious clones of three GGVA isolates—GGVA-17YM1 [8] and GGVA-76 and GGVA-93 [16]—all induced upward leaf curling and stunting symptoms in *N. benthamiana* plants, but grapevine (cultivars Colombard, Salt Creek, Cabernet Sauvignon, and Vaccarèse) plants infected with GGVA-76 or GGVA-93 did not display any obvious symptoms [16]. Therefore, our knowledge of the pathogenicity of GGVA to grapevine plants remains largely unclear.

In this study, we constructed an infectious clone containing the full genome of GGVA^QN^ to investigate its pathogenicity. *N. benthamiana* plants that were systemically infected with GGVA^QN^ showed symptoms of upward leaf curling and chlorotic mottling (Figure 2a), which were somewhat different from the upward leaf curling and stunting symptoms observed by Sun et al. [8] and Kuo et al. [16]. GGVA^QN^ shares 99.52%, 97.32%, and 98.51% sequence identity with GGVA-17YM1, GGVA-76, and GGVA-93, respectively. The inconsistency may not be due to the sequence differences between GGVA^QN^ and the three GGVA isolates; rather, other factors, such as the different ages of the *N. benthamiana* plants used for inoculation and fertilization, may be responsible.

The pathogenicity of GGVA^QN^ on grapevine was also investigated using in vitro-grown ‘Red Globe’ rooted plantlets. Upon GGVA^QN^ infection, all ‘Red Globe’ plants exhibited symptoms including chlorotic mottling, downward curling, necrosis of the leaf margins, and interveinal yellowing 2 months after inoculation and transplanting (Figure 3b and Figure 4a). The in vitro-grown ‘Red Globe’-rooted plantlets used for inoculation assays were negative for GGVA, GLRaV-4 and its strains GLRaV-5, GLRaV-6, GLRaV-9, GLRaV-De, and GLRaV-Pr, and another 17 viruses reported in China. Therefore, the symptoms of ‘Red Globe’ plants were closely associated with GGVA^QN^ infection. Our results differ from those reported by Kuo et al. [16], where neither GGVA-76 nor GGVA-93 induced obvious symptoms on grapevines ‘Colombard’, ‘Salt Creek’, ‘Cabernet Sauvignon’ and ‘Vaccarèse’ plants, although the virological condition of these plants was unclear. Lovato et al. [12] reported that grapevine plants infiltrated with the infectious clone of grapevine Algerian latent virus exhibited different symptoms in different cultivars. Therefore, the inconsistency may be due to the different grapevine cultivars used in the two studies. Future research investigating the pathogenicity of GGVA^QN^ in different grapevine cultivars is warranted.

One important factor that hinders the research on viruses of woody perennial plants, including grapevine, is the lack of a reliable experimental system for delivering infectious clones. Two main approaches, vacuum-agroinfiltration [2,9,16] and agro-drenching [10,15,18], are currently used to deliver infectious clones into grapevine plants. However, they are complex and time-consuming, and the achieved rate of infection is relatively low. In this study, we directly introduced *A. tumefaciens* cells carrying the infectious clone pXT-GGVA^QN^ into roots of grapevine plantlets by syringe followed by transplanting the plantlets into pots, and all surviving plants (15 out of 18) were systemically infected with GGVA^QN^. Therefore, we provide a very simple, quick, and efficient method for delivering infectious clones into grapevine plants with no vacuuming operation, taking only a few minutes to complete the infiltration, and resulting in a 100% infection rate. To our knowledge, the experimental protocol reported herein is the most effective system for achieving grapevine infection with infectious clones of a virus, potentially facilitating grapevine virology research.

In conclusion, an infectious clone of GGVA^QN^ was constructed, and its pathogenicity in *N. benthamiana* and grapevine plants was investigated. The results presented here provide important insights into GGVA pathogenesis as well as a very simple and highly effective method for launching grapevine infection with the infectious clone of a virus.

## 4. Materials and Methods

### 4.1. Plant Materials and Growth Conditions

Grapevine cv. Red Globe in vitro-grown rooted plantlets were previously obtained from treatments of thermotherapy combined with meristem tip culture and kept in our laboratory. The virological condition of these plantlets was investigated three times and was negative for GGVA and other viruses reported in China, including GLRaV-1, GLRaV-2, GLRaV-3, GLRaV-4 (including GLRaV-4 strains GLRaV-5, GLRaV-6, and GLRaV-9, GLRaV-De, and GLRaV-Pr), GLRaV-7, GLRaV-13, grapevine rupestris stem pitting-associated virus (GRSPaV), grapevine fleck virus (GFkV), grapevine fanleaf virus (GFLV), grapevine virus A (GVA), GVB, GVE, grapevine Pinot gris virus, grapevine fabavirus (GFabV), grapevine red blotch virus (GRBV), grapevine Syrah virus-1 (GSyV-1), grapevine berry inner necrosis virus (GINV), and grapevine red globe virus (GRGV).

In vitro-grown rooted grapevine plantlets were grown on woody plant medium supplemented with 30 mg mL^−1^ sucrose and 1 mg mL^−1^ indole-3-butyric acid. *N. benthamiana* plants were grown in individual pots containing commercial soil, and 6-week-old plants were used. All plant materials were grown at 26–31 °C with a 16-h light regime.

### 4.2. Construction of Infectious Clone of GGVA

The full genome of the GGVA^QN^ isolate was PCR amplified from a sample collected from a grapevine ‘Queen Nina’ plant in Guangxi Province in Southwest China using a TOPO-Blunt Cloning Kit (Vazyme, Nanjing, China) with the primer pair GGVA-1678-F/GGVA-1678-R previously described [8]. The PCR products were cloned into the pCE3 Blunt Vector provided by the kit, and the recombinant plasmid pCE3-GGVA^QN^ was used as the template to construct the infectious clone of GGVA.

Construction of the infectious clone of GGVA^QN^ was performed essentially as described previously [16], with modifications. A fragment (the linearized pXT1 without the sequence of the 35S promoter, multiple cloning site region, ribozyme, and NOS terminator) (Figure 1) was amplified using the binary vector pXT1 as template with primer pair F27/R27. Two fragments, each covering 0.6× viral genome sequence, were amplified from pCE3-GGVA^QN^ using primer pairs F25/R25 and F26/R26, with F25 and R26 containing a 30-bp sequence homologous to the T-DNA right border (RB) and left border (LB) on pXT1, respectively. The three fragments were assembled into circular plasmids using NEBuilder HiFi DNA Assembly Master Mix (New England Biolabs, Ipswich, MA, USA). Positive clones were identified by *Hind* III digestion (resulting in a 7197-bp fragment) and PCR with the primer pairs F25/R25 and F26/R26 for amplifying fragment 1 (1886 bp) and fragment 2 (1934 bp), respectively. Three positive clones were then sequenced to verify the correctness of the GGVA genome sequence. The recombinant plasmid pXT-GGVA^QN^ was delivered into competent *A. tumefaciens* strain GV3101 (Weidi Biotechnology, Shanghai, China) using the freeze–thawing method according to the manufacturer’s instructions.

### 4.3. Inoculation

*A. tumefaciens* GV3101 carrying the infectious clone pXT-GGVA^QN^ was cultured to an optical density of 1.0 at 600 nm. *Agrobacterium* cells were harvested by centrifugation and resuspended in the infiltration buffer (10 mM MES/NaOH pH 5.8, 10 mM MgCl_2_, 150 µM acetosyringone). Agroinfiltration of *N. benthamiana* plants was performed essentially as described previously [20].

For inoculation of in vitro-grown grapevine plantlets, rooted ‘Red Globe’ plantlets were removed from the culture jar and washed to remove the agar adhering to their base. Plantlets with pruned roots and leaves were placed on moist filter paper. A 0.5-mL aliquot of *A. tumefaciens* GV3101 cells carrying pXT-GGVA^QN^ (for each grapevine plantlet) was introduced into the main root bark with a syringe, and the plantlet was transplanted into a pot (10 cm diameter) containing commercial soil (Figure 3a). These pots were placed in plates filled with a shallow layer of water. About 3 days after transplanting, new buds were observed, indicating that they had survived. We used 8–13 plants, or plantlets, for each treatment in each experiment.

All inoculated plants were fertilized once a week with water-soluble fertilizer (Peters Professional).

### 4.4. RT-PCR and PCR

The virological condition of the in vitro-grown grapevine plantlets was detected by RT-PCR and PCR, and the presence of GGVA in inoculated plants was assessed by PCR.

Primers used in the RT-PCR and PCR assays are listed in Supplementary Table S1. Primer pairs F6/R6, F19/R19, F20/R20, F25/R25, F26/R26, and F27/R27 were designed in this study, and others were described previously [21,22,23,24,25,26,27,28,29,30,31,32,33]. RNA and DNA were extracted from *N. benthamiana* leaves, or phloem scrapings of grapevine petioles or leaves of in vitro-grown grapevine plantlets using the cetyltrimethylammonium bromide method [34]. The synthesis of cDNA (at 37 °C) was primed with a mixture of random primers using 500 ng of total RNA with HiScript III 1st Strand cDNA Synthesis Kit (Vazyme, Nanjing, China). A 1-μL aliquot of cDNA or DNA was used as a template in a 10 μL PCR, and PCR amplification was conducted as follows: DNA denaturation at 95 °C for 5 min; 35 cycles at 95 °C for 30 s, 52–60 °C (depending on the specific primers used) for 30 s, and 72 °C for 30 s, and a final extension step at 72 °C for 5 min. A negative control (ddH_2_O instead of cDNA or DNA) was included in the PCR assays. Positive PCR products were randomly selected for sequencing to confirm their identity.

### 4.5. Western Blot Analysis

Plant protein extraction and Western blot analysis were performed as detailed previously [20]. The dilution of anti-V1 antiserum (noncommercial) used in the Western blot analysis was 1:2000. The hybridization signals were detected with High-sig ECL Western Blotting Substrate (Beyotime, Shanghai, China) according to the manufacturer’s instructions. Coomassie brilliant blue staining confirmed equal protein contents in each lane.

## Data Availability

Data are contained within the article and Supplementary Materials.

## Supplementary Materials

The following supporting information can be downloaded at: https://www.mdpi.com/article/10.3390/plants13121601/s1, Table S1: Primers used in this study; Figure S1: Sequence alignment and phylogenetic tree analysis of GGVA isolates. (a) Sequence alignment between GGVA^QN^ and the isolate (GGVA^ML^) obtained from grapevine ‘Marselan’. (b) Phylogenetic tree based on the whole genome sequences of GGVA isolates from GenBank. Evolutionary analyses were conducted in MEGA7 (www.megasoftware.net). The evolutionary history was inferred using the neighbor-joining method. The bootstrap consensus tree inferred from 2000 replicates is taken to represent the evolutionary history of the taxa analyzed. ● indicates GGVA^QN^ and GGVA^ML^. ■ and ♦ indicate the isolates reported by Sun et al. [8] and Kuo et al. [16], respectively; Figure S2: Investigation of the virological condition of in vitro-grown grapevine ‘Red Globe’ plantlets. N, negative control with ddH_2_O instead of DNA or cDNA.
