# Supplementary material for: Construction of an Infectious DNA Clone of Grapevine Geminivirus A Isolate GN and Its Biological Activity in Plants Analyzed Using an Efficient and Simple Inoculation Method
Source: Plants (Basel). 2024 Jun 8;13(12):1601. doi: 10.3390/plants13121601 (PMC11207333; doi:10.3390/plants13121601)
Supplement: Supplementary file 1 [file plants-13-01601-s001.zip › Supplementary Materials.pdf]

Table S1. Primers used in this study

Primer	Sequences (5' →3')	Gene	Size (bp)	References
F1	GGTTGGGTAAGCCGTTGAGAGCTCT	GLRaV-1 *HSP70*	637	[22]
R1	TAAGTTCCTCAGCACCTTCTACGA			
F2	TTTGCGAAGAAAGAAATCGTTAAGATGAGG	GLRaV-2 *p24*	711	[29]
R2	TACGTCAGGTAGATACACCCACGTTCGA			
F3	GCCCGCATCGATATGGACCTATCGTTTATT	GLRaV-3 *p19.7*	540	[29]
R3	GCCGTCGACTTATAGTGCTCCGCAACAAA			
F4	ACATTCTCCACCTTGTGCTTTT	GLRaV-4 *HSP70*	319	[33]
R4	CATACAAGCGAGTGCAATTACA			
F5	AACACTCTGCTTTTCTGCTGGCA	GLRaV-5 *p70*	272	[33]
R5	TCTCCAGAAGACGGACCAATGTAA			
F6	GTTCTCCACGTTGTGCTTCTCC	GLRaV-6 *HSP70*	480	Designed in this study
R6	GGTTCATTCACCACTGCTTGAACC			
F7	CGAAGGTTTGCTATACGTCAACCC	GLRaV-7 *p61*	518	[32]
R7	TGAACAAGTGACGCCGACGA			
F8	CGGCATAAGAAAAGATGGCAC	GLRaV-9 *HSP70*	393	[33]
R8	TCATTCACCACTGCTTGAAC			
F9	ATCAGGTCGTGAACCAAGCAGG	GLRaV-13 *HSP70h*	253	[31]
R9	TAAGACCAGGTTCGACCGTTGG			
F10	AAAGAYACCCCSGTTAGGGGWTTTC	GLRaV-De *CP*	1306	[25]
R10	CCRTTATTWGTAGCTACWACATTRCCYA			
F11	AAAGAYACCCCSGTTAGGGGWTTTC	GLRaV-Pr *CP*	1267	[25]
R11	CCRTTATTWGTAGCTACWACATTRCCYA			
F12	CCWGACYTMTCYYTRCCAAG	GFLV *CP*	450	[23]
R12	GGYTTRCACAARACDCGGAG			
F13	ATTGCCCGCAACCCTCGTGTAAGC	GFkV *CP*	366	[22]
R13	CGGAGTCCTTGATGGTGGGGTTGA			
F14	GACAAATGGCACACTACG	GVA *CP*	429	[22]
R14	AAGCCTGACCTAGTCATCTTGG			
F15	GTGTACGAGACAATAAGCAAGCA	GVB partial ORF3,	722	[24]
R15	GTAGCCCTTCGTTTAGCCGCACT	ORF4, IR and ORF5		
F16	AGGCGAATCAAGTACTTCATG	GPGV *CP*	340	[22]
R16	GGTGCTTCTTGTATATATTAGTATGC			
F17	ATGTCGATMAGRCAGGAATTG	GINV *CP*	585	[21]
R17	CATAGTAAAAGCACCCTCGCT			
F18	CGTCACTGCTCTGATGTTGGTAG	GRSPaV *CP*	327	[22]
R18	AGGCGATATTAGCAACCATCTCAG			
F19	GGAAATAACATTCCCAGGAAG	GFabV *polyprotein*	313	Designed in this study
R19	TCCTGCTGTCAAATTTCATTC			
F20	ATGGACTTCAATCCGAGAAAGAGG	GGVA *V1*	685	Designed in this study
R20	GGCCTCCATACTTAATATCAGAGTAGG			

F21	AGCGGAAGCATGATTGAGACATTGACG	GRBV *CP*	231	[30]
R21	AACGTATGTCCACTTGCAGAAGCCGC			
F22	CACCCCTTCTCCCCATGTGAC	GRGV *CP*	441	[26]
R22	ACAGCTGGGGCTTCAGGGTT			
F23	CAAGCCATCCGTGCATCTGG	GSyV-1	297	[27]
R23	GCCGATTTGGAACCCGATGG	*methyltransferase*		
F24	GTGGGTGAACCACTCAAGGT	GVE *CP*	478	[28]
R24	AGACCACTTGCGGCTCTTTA			
F25	GTTTAAACTGAAGGCGGGAAACGACAAT<br>CTAGACTCTTTGGATATATGGCACTGAGG	GGVA Fragment 1	1886	Designed in this study
R25	GAAGGATCAATTGCCAGTGCAATTG			
F26	GATACAATTGCACTGGCAATTGATCC	GGVA Fragment 2	1934	Designed in this study
R26	TAACACATTGCGGACGTTTTTAATGTAC<br>TGCGTCATCATGGTATCCCTTGTCAGG			
F27	CAGTACATTAAAAACGTCCGCAATGTG	linearized pXT1	3466	Designed in this study
R27	AGATTGTCGTTTCCCGCCTTCAG			
F28	CCCCATGCTATCCTTCG	Grapevine *actin*	216	[22]
R28	AGGCAGCTCATAGTTCTTCTC			

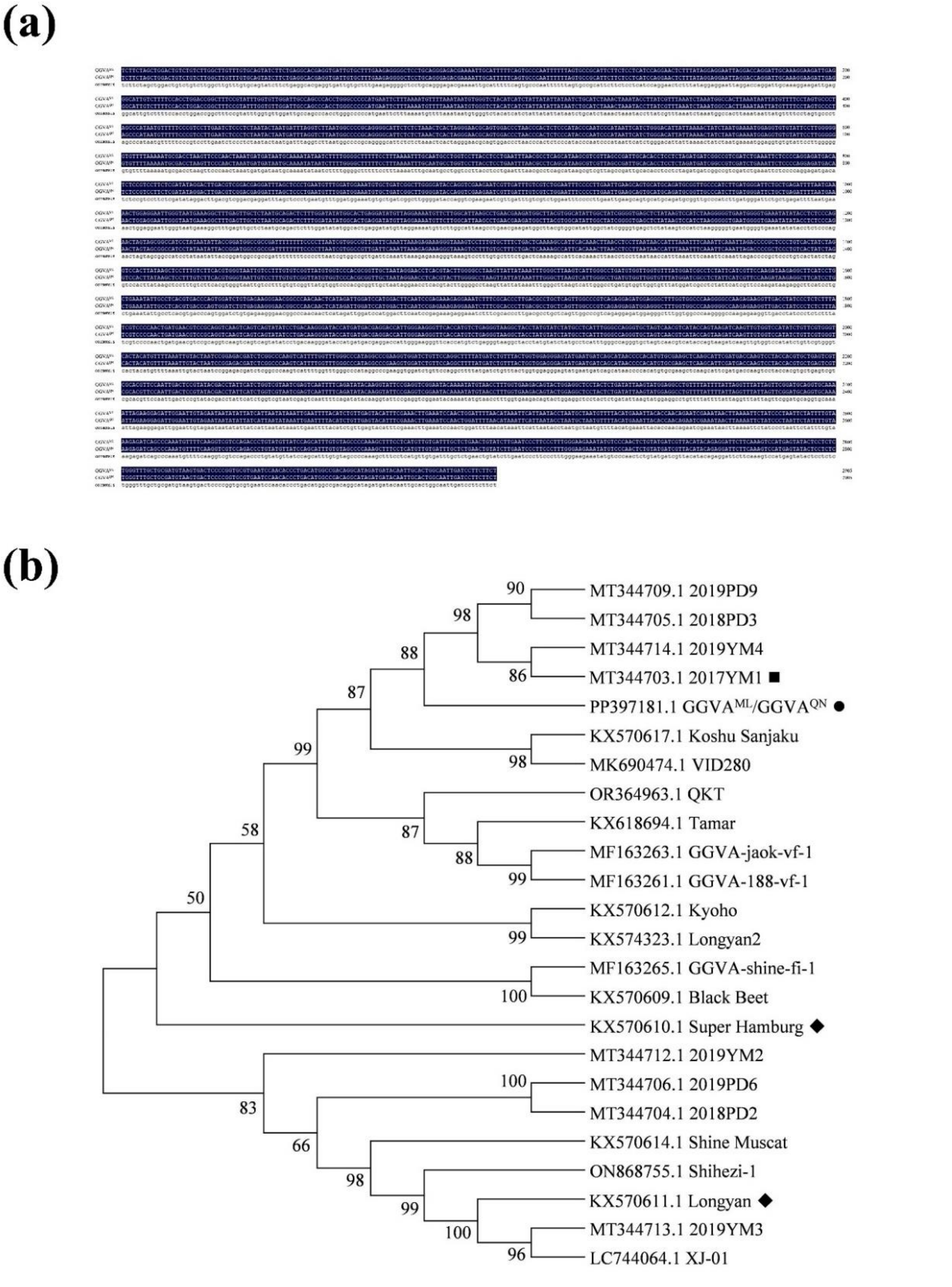

Figure S1. Sequence alignment and phylogenetic tree analysis of GGVA isolates. (a) Sequence alignment between GGVA^{QN} and the isolate (GGVA^{ML}) obtained from grapevine 'Marselan'. (b) Phylogenetic tree based on the whole genome sequences of GGVA isolates from GenBank. Evolutionary analyses were conducted in MEGA7 (www.megasoftware.net). The evolutionary history was inferred using the neighbor-joining method. The bootstrap consensus tree inferred from 2000 replicates is taken to represent the evolutionary history of the taxa analyzed. ● indicates GGVA^{QN} and GGVA^{ML}. ■ and ◆ indicate the isolates reported by Sun et al. [8] and Kuo et al. [16], respectively.

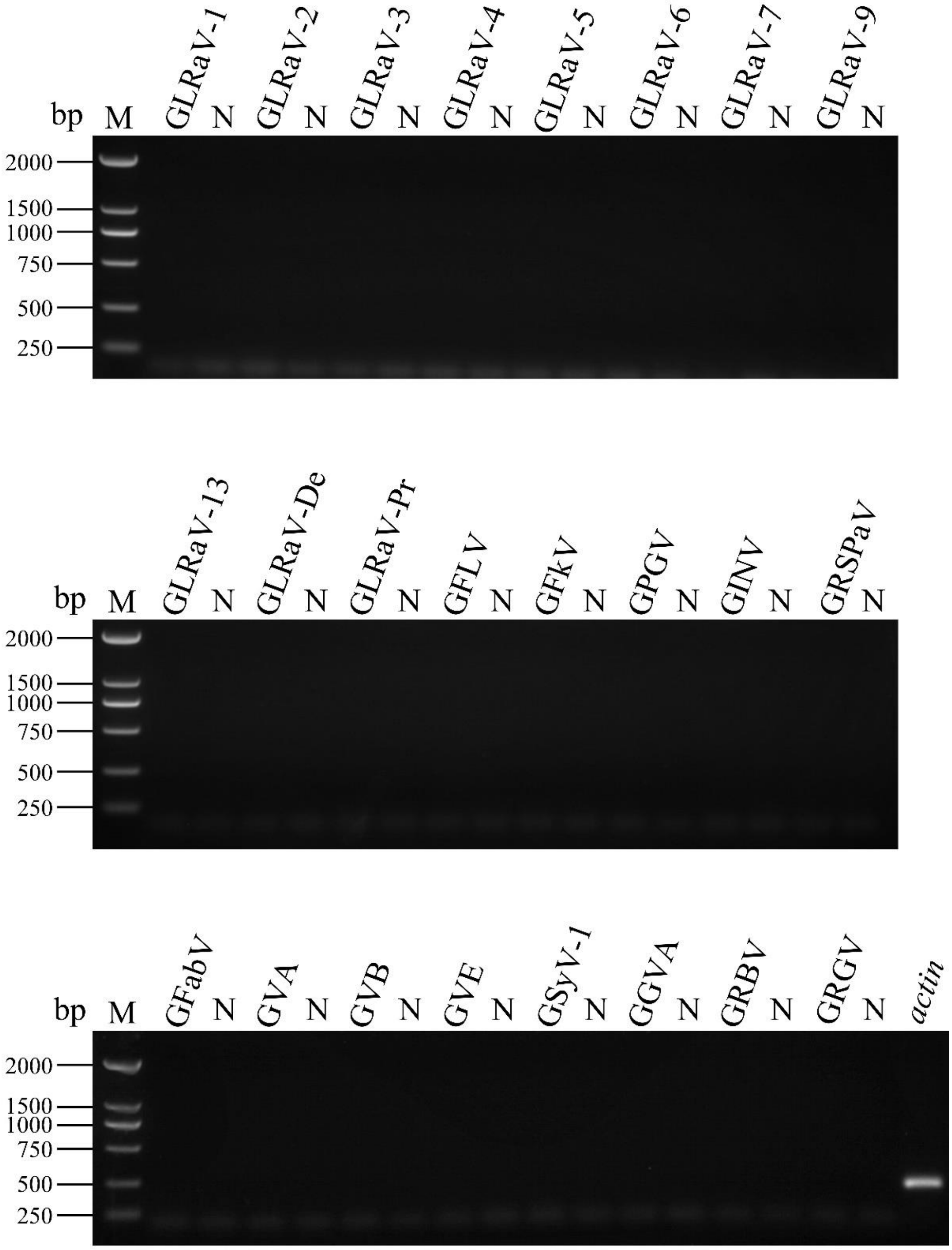

Figure S2. Investigation of the virological condition of in vitro-grown grapevine 'Red Globe' plantlets. N, negative control with ddH2O instead of DNA or cDNA.
